# Comparative analysis among the small RNA populations of source, sink and conductive tissues in two different plant-virus pathosystems

**DOI:** 10.1186/s12864-015-1327-5

**Published:** 2015-02-22

**Authors:** Mari Carmen Herranz, Jose Antonio Navarro, Evelien Sommen, Vicente Pallas

**Affiliations:** Instituto de Biología Celular y Molecular de Plantas, Universidad Politécnica de Valencia-Consejo Superior de Investigaciones Científicas, Campus UPV, CPI 8E, Avda. Ingeniero Fausto Elio s/n, Valencia, 46022 Spain

**Keywords:** Melon, Cucumber, Virus, Deep-sequencing, Small RNAs, Phloem

## Abstract

**Background:**

In plants, RNA silencing plays a fundamental role as defence mechanism against viruses. During last years deep-sequencing technology has allowed to analyze the sRNA profile of a large variety of virus-infected tissues. Nevertheless, the majority of these studies have been restricted to a unique tissue and no comparative analysis between phloem and source/sink tissues has been conducted. In the present work, we compared the sRNA populations of source, sink and conductive (phloem) tissues in two different plant virus pathosystems. We chose two cucurbit species infected with two viruses very different in genome organization and replication strategy; *Melon necrotic spot virus* (MNSV) and *Prunus necrotic ringspot virus* (PNRSV).

**Results:**

Our findings showed, in both systems, an increase of the 21-nt total sRNAs together with a decrease of those with a size of 24-nt in all the infected tissues, except for the phloem where the ratio of 21/24-nt sRNA species remained constant. Comparing the vsRNAs, both PNRSV- and MNSV-infected plants share the same vsRNA size distribution in all the analyzed tissues. Similar accumulation levels of sense and antisense vsRNAs were observed in both systems except for roots that showed a prevalence of (+) vsRNAs in both pathosystems. Additionally, the presence of overrepresented discrete sites along the viral genome, hot spots, were identified and validated by stem-loop RT-PCR. Despite that in PNRSV-infected plants the presence of vsRNAs was scarce both viruses modulated the host sRNA profile.

**Conclusions:**

We compare for the first time the sRNA profile of four different tissues, including source, sink and conductive (phloem) tissues, in two plant-virus pathosystems. Our results indicate that antiviral silencing machinery in melon and cucumber acts mainly through DCL4. Upon infection, the total sRNA pattern in phloem remains unchanged in contrast to the rest of the analyzed tissues indicating a certain tissue-tropism to this polulation. Independently of the accumulation level of the vsRNAs both viruses were able to modulate the host sRNA pattern.

**Electronic supplementary material:**

The online version of this article (doi:10.1186/s12864-015-1327-5) contains supplementary material, which is available to authorized users.

## Background

In most eukaryotes RNA silencing is an essential mechanism which controls gene expression in a wide range of biological processes. In addition, RNA silencing plays an important role as defence against viruses in plants, invertebrates and possibly also in mammals [1-4]. Double-stranded RNA (dsRNA) is the factor triggering the process and upon infection the host silencing machinery is induced by dsRNAs generated during viral replication or secondary structures derived from the pathogen genome [5-7]. These viral dsRNAs are processed by RNase III enzymes belonging to the Dicer family (Dicer-like or DCL in plants). In *Arabidopsis thaliana,* four classes of DCL enzymes (DCL1 to DCL4) have been reported, DCL1 mainly contributes to the production of miRNAs [8] and DCL4, DCL2 and DCL3 are involved in the processing of viral genomes yielding viral-sRNAs (vsRNAs) of 21-, 22- and 24-nt, respectively [9-11]. In infected plants, hierarchical roles of DCL4 and DCL2 have been established; 21-nt vsRNAs are the most abundant class followed by 22-nt vsRNAs [12-16]. Subsequent loading of vsRNAs to Argonaute (AGO)-containing complexes or RISCs directs the degradation of both genomic and subgenomic viral RNAs [17,18]. In plants, this antiviral silencing response can be amplified by cellular RNA-dependent RNA polymerases (RDRs) which use as templates cleaved viral RNAs to generate new dsRNA substrates for Dicer enzymes [19].

In plants, a series of grafting experiments demonstrated that RNA silencing has a systemic nature and that the long-distance transmission of the putative sequence-specific signaling molecule must be transported through the phloem [20].

Little is known about the nature of the systemic signal that mediates the process and how it is transmitted from the site of initiation to the rest of the plant. However, the detection of sRNAs in the phloem of different plant species [21-24] as well as the identification of phloem RNA binding proteins with capacity to bind and translocate sRNAs [21,25] have led to the assumption that the systemic signal must be a ribonucleoprotein complex. In addition, Tournier et al. [26] demonstrated that the silencing signal is transported from source to sink tissues following the direction of phloem flow. Whereas some lines of evidence have indicated a role for sRNAs with different sizes in long-distance transmission of RNA silencing [27-29], other studies have suggested that the signal could be either a sRNA precursor or produced from dsRNA by a DCL-independent mechanism [30].

During last years deep-sequencing analyses in different plant/virus systems have allowed to study the vsRNA profile of a high variety of infected tissues [7,31-42]. Nevertheless, with very few exceptions [32,35] this type of studies have been restricted to a unique tissue and no comparisons among phloem and source/sink tissues have been made. Therefore, the specific aims of this work were: 1) to compare the populations of viral-derived sRNAs among source, sink and conductive (phloem) tissues and 2) to compare these populations in two different pathosystems sharing cucurbit species as a host. To do that, we selected two plant hosts infected with viruses very different in genome organization and replication strategy. Thus, inoculated cotyledon as a source, root and symptomatic primary leaf as recipient tissues and phloem sap from melon and cucumber plants infected with *Melon necrotic spot virus* (MNSV) and *Prunus necrotic ringspot virus* (PNRSV), respectively, were analyzed.

*Melon necrotic ringspot virus* (MNSV) is a plant virus belonging to the genus *Carmovirus* within the family *Tombusviridae* [43,44] which is present in cucurbit crops worldwide. The MNSV genome is a single-stranded RNA molecule of 4.3 kb with positive polarity encoding at least five different proteins [44,45]. The open reading frame (ORF) at the 5′ end terminates in an amber codon yielding two proteins involved in replication, p29 and p89. Cell-to-cell viral movement is supported by two proteins, p7A and p7B, encoded by two small centrally-located ORFs [46-49]. The ORF at the 3′end encodes the coat protein (CP) p42 which is also involved in systemic transport of the virus and is a symptom determinant [50]. By contrast, *Prunus necrotic ringspot virus* (PNRSV) is a positive single-stranded RNA virus, member of the genus Ilarvirus in the family *Bromoviridae* and with a tripartite genome. RNAs 1 and 2 encode the replicase subunits P1 and P2. RNA 3 is bicistronic and contains the coding sequences of the putative movement protein (MP) gene and the CP gene. CP synthesis occurs via a subgenomic monocistronic mRNA (RNA 4) [51,52].

Our findings showed, in both systems, an increase of the 21-nt total sRNAs together with a decrease of those with a size of 24-nt in all the infected tissues, except for the phloem where the ratio of 21/24-nt sRNAs remained constant. Comparing the vsRNAs, both PNRSV and MNSV infected plants share the same vsRNA size distribution in all the analyzed tissues. Although in PNRSV-infected plants the percentage of vsRNAs was one hundred times less than that observed for the MNSV- infected plants, both viruses were able to modulate the host sRNA profile. In addition, similar accumulation levels of sense and antisense vsRNAs were observed in both systems except for roots that showed a prevalence of (+) vsRNAs in both pathosystems. The biological significance of these results is discussed.

## Results

To determine virus-induced changes in the sRNA profiles of cotyledon, leaf, root and phloem samples of MNSV- and PNRSV-infected or mock-inoculated melon and cucumber plants, we conducted Solexa sRNA profiling (HiSeq2000) and bioinformatic analysis of the sequence. The results were further confirmed by polyacrylamide gels stained with silver nitrate (PAGE-SN). A total of 16 sRNA libraries were constructed generating between 6 to 15 million reads each (Table 1). As it is indicated in Additional file 1: Table S1 most of the inserts found have a length ranging from 18 to 26 bases.

**Table 1:** **Viruses, host plants and tissues used for construction of sRNA libraries**

**Library**	**Host**	**Tissue**	**Virus**	**Number of reads**
1	Melon	Cotyledon	Healthy	9,715,225
2	Melon	Cotyledon	MNSV	9,607,706
3	Cucumber	Cotyledon	Healthy	10,404,176
4	Cucumber	Cotyledon	PNRSV	11,495,525
5	Melon	Leaf	Healthy	10,301,642
6	Melon	Leaf	MNSV	10,573,642
7	Cucumber	Leaf	Healthy	8,295,114
8	Cucumber	Leaf	PNRSV	12,859,274
9	Melon	Root	Healthy	9,086,537
10	Melon	Root	MNSV	9,552,572
11	Cucumber	Root	Healthy	14,212,925
12	Cucumber	Root	PNRSV	8,851,247
13	Melon	Phloem	Healthy	12,129,387
14	Melon	Phloem	MNSV	13,108,602
15	Cucumber	Phloem	Healthy	7,117,664
16	Cucumber	Phloem	PNRSV	6,179,826

To confirm that our virus-infected samples were indeed infected and to compare virus accumulation in melon and cucumber, respectively, we performed Northern blot analysis of total RNA from cotyledon, leaf, root and phloem samples of MNSV and PNRSV-infected plants using a virus-specific probe (Additional file 2: Figure S1). Analysis of serial dilutions of each infected sample by dot-blot (Additional file 3: Figure S2) and quantification of the signal using the Java image processing programe ImageJ indicated similar virus concentrations in leaves and the highest difference in cotyledons with a ratio of 7 considering the higher dilution of the virus (Additional file 4: Table S2).

### The total sRNA size profile differs in healthy and MNSV infected melon plants

**Figure 1:**
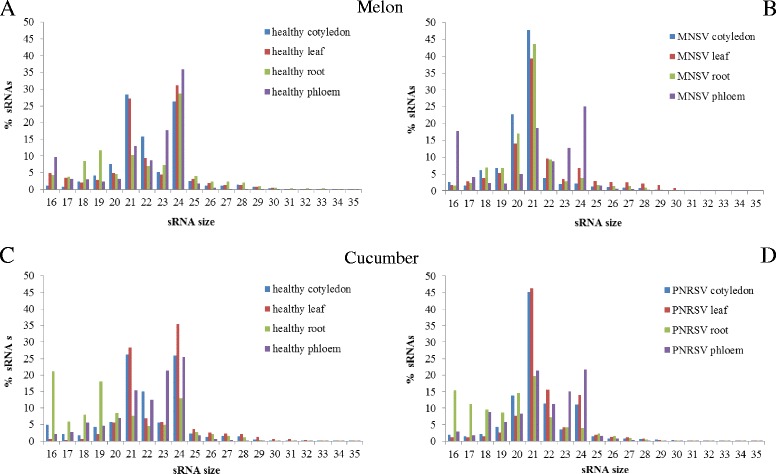
**Size-distribution of total sRNAs (from 16 to 35 nt) in the sixteen sequenced libraries.** The different tissues are represented with colors: cotyledon (blue), leaf (red), root (green) and phloem (violet). Graphs **A** and **C** show the profiles in healthy plants of melon and cucumber respectively, **B**, in MNSV-infected plants and **D**, in PNRSV-infected plants.

The total sRNA profile was compared between healthy and MNSV-infected melon samples (Figure 1). While the highest amount of sRNAs in healthy leaf and cotyledon tissues were 21- and 24-nt in length representing around 60% in total, the most abundant sRNAs in root and phloem tissue were those with a lengh of 24-nt length (around 30% of the total sRNAs) (Figure 1A).

**Figure 2:**
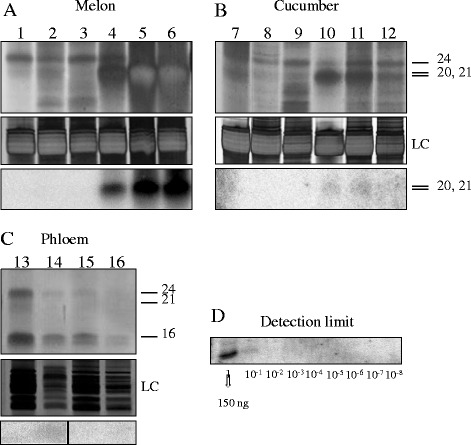
**Analysis of the total sRNA population on silver nitrate-stained polyacrylamide gels and isotopic detection of the viral sRNAs (vsRNAs) by Northern blot.** (**A**, **B** and **C** upper pannels) Separation of the total sRNAs in polyacrylamide/Urea gels stained with silver nitrate. **(A)** Melon tissues; healthy leaf, cotyledon and root (lanes 1, 2 and 3) and MNSV-infected leaf, cotyledon and root (lanes 4, 5 and 6). **(B)** Cucumber tissues; healthy leaf, cotyledon and root (lanes 7, 8 and 9) and PNRSV-infected leaf, cotyledon and root (lanes 10, 11 and 12). **(C)** Melon (lane 13) and cucumber (lane 15) healthy phloem, MNSV (lane 14) and PNRSV (lane 16) infected phloem. (**A**, **B** and **C** lower pannels) Northern blot analysis to detect MNSV (A y C) and PNRSV (B y C) derived sRNAs in the different tissues. **(D)**. Detection limit of the PNRSV probe. Serial dilutions of an artificial PNRSV sRNA were blotted onto a nylon membrane and hybridized with the isotopic PNRSV probe. LC: Loading control.

In infected melon cotyledon, leaf and root tissues, a clear increase (around 50, 40 and 45%, respectively) of the 21-nt sRNAs was observed whereas the percentage of the population with a length of 24 nucleotides diminished considerably (around 5% and below). Compared to the PAGE-SN (Figure 2A upper panel), the increase of the 21-nt species was much more evident being higher in infected cotyledon and root (lanes 5 and 6) than in leaf (lane 4). A smear under the 21-nt band could also be noticed representing the 20-nt sRNAs (Figure 2A, lanes 4, 5 and 6), the second size more abundant in MNSV infected leaf, cotyledon and root according to the deep-sequencing analysis (Figure 1B).

The sRNA profile of phloem sap was considerably different from the profile of the other tissues. As it is shown in Figure 1 the majority of sRNAs in healthy and infected phloem have a length of 24-nt (35%). The band corresponding to this size is clearly visible in the PAGE-SN (Figure 2C, lanes 13 and 14). Moreover, in both the infected and healthy plants, a higher amount of small RNAs with a 16-nt size was observed (Figure 1 and Figure 2C, lanes 13 and 14). In contrast to the other tissues, the 21-nt sRNA population did not significantly increase upon infection in phloem sap. In addition, in this tissue, the 21/24 ratio is maintained regardless of whether the plant is infected or not (Additional file 5: Figure S3A).

### The sRNA size profile differs in healthy and PNRSV-infected cucumber plants

Analysis of the sRNA profile in healthy and PNRSV-infected cucumber plants revealed that the total sRNAs size distribution pattern was very similar to that observed for melon plants. Compared with healthy tissues, infected cotyledon and leaf samples exhibited increased levels of 21-nt sRNAs (from around 27% to 45% in both tissues) together with a lower amount of 24-nt (from 25% to 12% in cotyledon and 35% to 14% in leaf) species (Figure 1C and 1D). It is noticeable that in infected root- although there is also an increase-the amount of sRNAs with a size of 21-nt was smaller (Figure 1D) and species with a size between 16 and 20-nt were much more represented in both healthy and infected root. This is also clearly visible in the PAGE-SN (Figure 2B, lanes 9 and 12) where a band pattern appears below the 21-nt size. As seen in cotyledons and leaves, virus infection produced a three-fold reduction of the sRNAs with a size of 24-nt (approximately 13% in mock-inoculated vs 4% in PNRSV-infected roots).

The sRNA profile of cucumber phloem was different from the profile of the other tissues. While phloem of healthy cucumber plants contained mostly sRNAs with a size of 24 (25%), 23 (21%) and 21 (15%) nucleotides (Figure 1C), the ratio 21/24-nt sRNAs remained constant in infected phloem tissue (Figure 1D and Additional file 5: Figure S3B). Although the signal was very weak, we could confirm the presence of the 24-nt sRNAs by polyacrylamide gel analysis (Figure 2C, lanes 15 and 16), the rest of the sizes were hardly visible. As for melon phloem tissue, a more intense double band at the bottom of the gel appeared as well.

### The percentage of vsRNAs in PNRSV infected cucumber plants is below 1%

**Figure 3:**
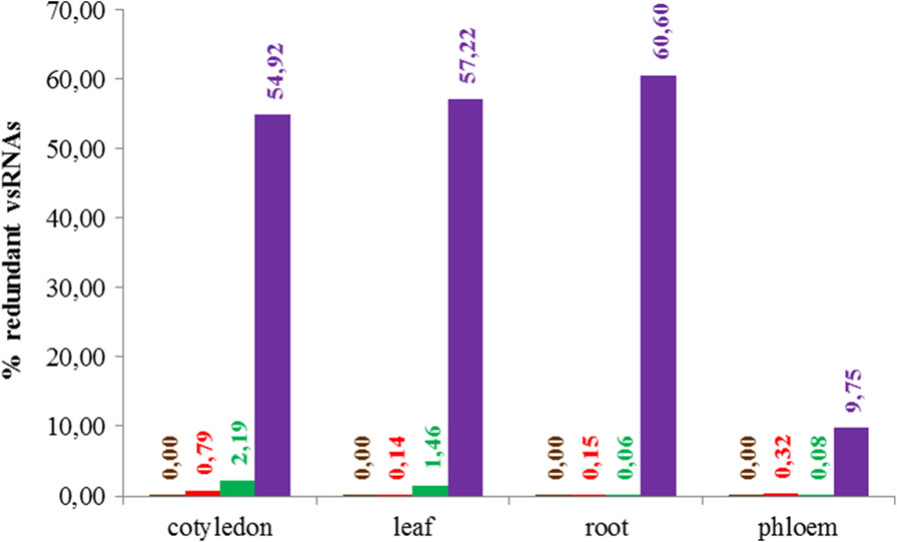
**Graphic representation of the percentage of redundant vsRNAs in all the tissues.** Normalized data for each library were represented. The same color depicts the four tissues from healthy cucumber (brown), PNRSV-infected cucumber (red), healthy melon (green) and MNSV-infected melon (violet).

In order to know which percentage of the total sRNAs represented the vsRNAs, a blast of each library against the corresponding viral genome was carried out. Figure 3 shows the results for each tissue in both virus/plant systems used in this study. Remarkably, the percentage of vsRNAs in cotyledons, leaves and roots of infected melon plants was more than 50% whereas in phloem the amount was around 10%. By contrast, the amount of viral sRNAs in all the analyzed tissues from infected cucumber plants was below 1% being even lower in leaf and root.

### vsRNAs are mainly 21 nucleotides in length

**Figure 4:**
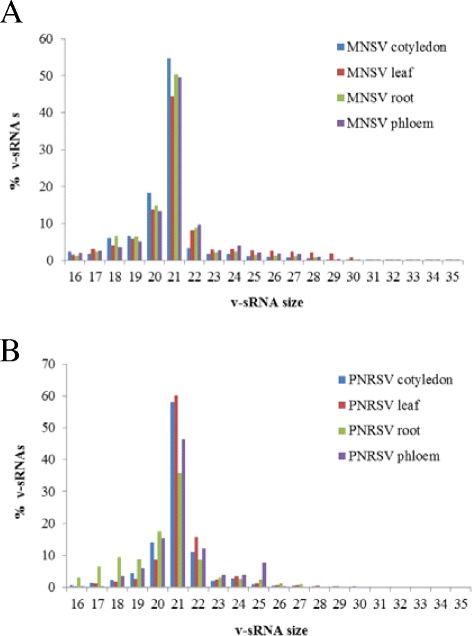
**Size distribution of MNSV and PNRSV derived sRNAs (from 16 to 35 nt) in the eight infected samples. (A)** Size distribution of MNSV derived sRNAs and **(B)** size distribution of PNRSV derived sRNAs. Normalized values for the different tissues are represented in colors: cotyledon (blue), leaf (red), root (green) and phloem (violet).

Size distribution analysis showed that the vsRNAs were predominately 21-nt in length for both MNSV and PNRSV in leaves, cotyledons, roots and phloem (Figure 4). In melon plants and all the tissues, about 50% of the vsRNAs had a length of 21-nt followed by approximately 15% with 20-nt. It has been reported that 20-nt sRNAs likely result from partial degradation of the 21-nt sRNAs, as they were undetectable in *dcl4* mutant *A. thaliana* plants infected with the carmovirus *Turnip crinkle virus* (TCV) [16]. Validation by isotopic hybridization using a specific riboprobe confirmed that the vsRNAs were predominantly 21 nucleotides in length (Figure 2A, lower panel), including in phloem where the hybridization signal was close to the detection limit (Figure 2C, lower panel).

In cucumber the results were similar, approximately 50% of the vsRNAs from PNRSV infected leaves, cotyledons, roots and phloem had a length of 21 nucleotides, however, they were hardly detected in infected cotyledon, leaf and root tissues (Figure 2B, lower panel). This low vsRNA signal confirms the low percentage of these species found in PNRSV-infected cucumber plants by deep-sequencing analysis.

Based on these two observations, we decided to test whether or not the concentration of vsRNAs in infected cucumber plants was under the detection limit analyzing serial dilutions of a 21-nt transcript with known concentration by hybridization with the isotopic riboprobe. With this approach we were able to detect up to 15 ng of the specific sRNA (Figure 2D). Considering the percentages of vsRNAs in all infected cucumber tissues, calculations were made with cotyledon which has the highest concentration representing 0.8% of the total RNA. 4 μg of sample containing thus 32 ng of vsRNAs were loaded into the gel and therefore, the amount of vsRNAs in the cucumber samples was close to the detection limit and clearly under this limit for the phloem samples. This result explains the weak or null signals obtained in the Northern blot analysis and validates the low percentage of vsRNAs observed in infected cucumber plants in comparison with the infected melon plants. Although it is reasonable to assume that accumulation levels of vsRNAs could correlate with the accumulation levels of viral RNAs this seems not to be the case since, as stated above, both pathosystems accumulated similar viral RNAs levels (Additional file 2: Figure S1).

### The antiviral silencing machinery acts mainly through DCL4 in this plant genus

Knowing that vsRNAs in melon and cucumber plants were mainly 21-nt in size, we decided to analyze expression levels of the different DCL transcripts by real-time qRT-PCR. A genome-wide analysis using protein BLAST search of the corresponding databases (http://www.phytozome.net and http://www.melonomics.net) identified the four homologues of *A. thaliana* DCL1 to 4 in both *C. sativus* and *C. melo* genome (melon accession numbers and cucumber locus names are provided in Methods section). Additional file 6: Figure S4 and Additional file 7: Figure S5 show the differential expression of DCL1 to 4 transcripts in different tissues of both melon and cucumber, respectively. As the expression of these enzymes has not been described in phloem to date, this tissue was excluded from the analysis. According to the results, DCL4 seemed to be the most important antiviral silencing enzyme as its expression level clearly increased in all the infected tissues and in both pathosystems. Regarding the other DCLs, the patterns that compared both hosts differed. Upon MNSV infection, we observed a significant increase in the expression level of the DCL3 transcript in infected cotyledon (Additional file 6: Figure S4). However in the PNRSV-infected cucumber plants, infection modulated the expression of DCL3 in leaves and roots, and also of DCL2 in cotyledon which led to higher levels of transcripts (Additional file 7: Figure S5).

### Mapping of vsRNAs against the viral genome suggests tissue-specific differences in the origin of the viral sRNAs

**Figure 5:**
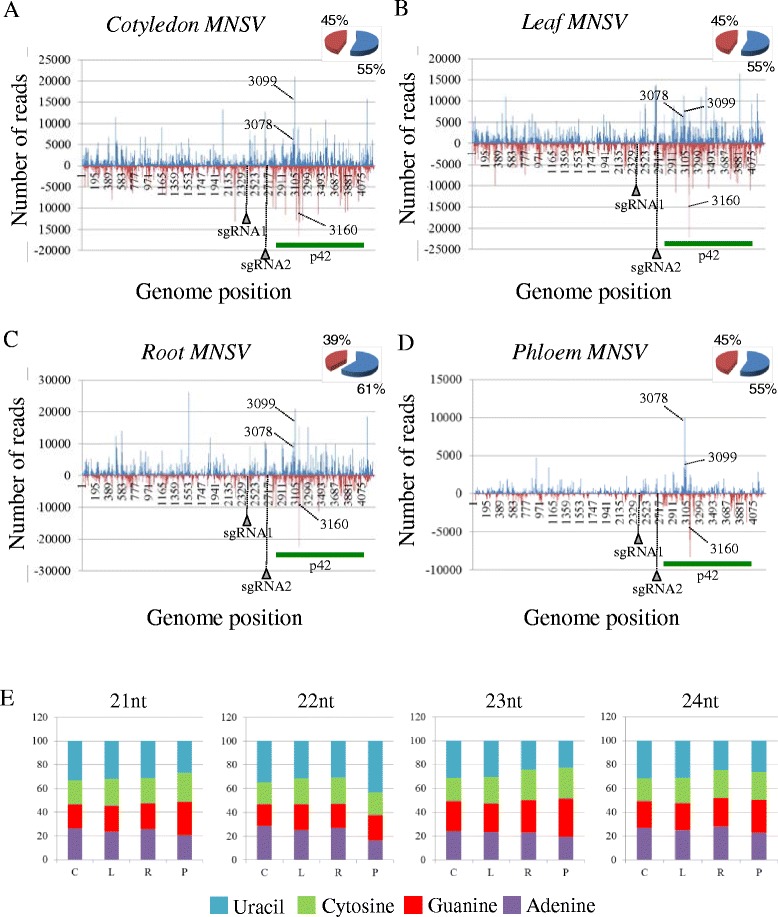
**Distribution of vsRNAs along the** ***Melon necrotic spot virus*** **(MNSV) genome in both positive and negative polarity and relative abundance of the four different 5′terminal nucleotides in the most representative species of vsRNAs.** **A**, **B**, **C** and **D** represent the distribution of vsRNAs along the MNSV genome in cotyledon, leaf, root and phloem respectively in both polarities; positive in blue and negative in red. Arrows indicate the 5′end of MNSV subgenomics RNAs (sgRNA1 and sgRNA2). The green bold line shows the location of the coat protein p42. Broken lines indicate the hotspots validated by stem-loop RT-PCR. Pie graphs (upper right corners in **A**, **B**, **C** and **D**) show the percentage of vsRNAs in each polarity. **(E)** Percentage of each 5′terminal nucleotides in all the tissues and for the most abundant vsRNAs species (21 to 24-nt).

**Figure 6:**
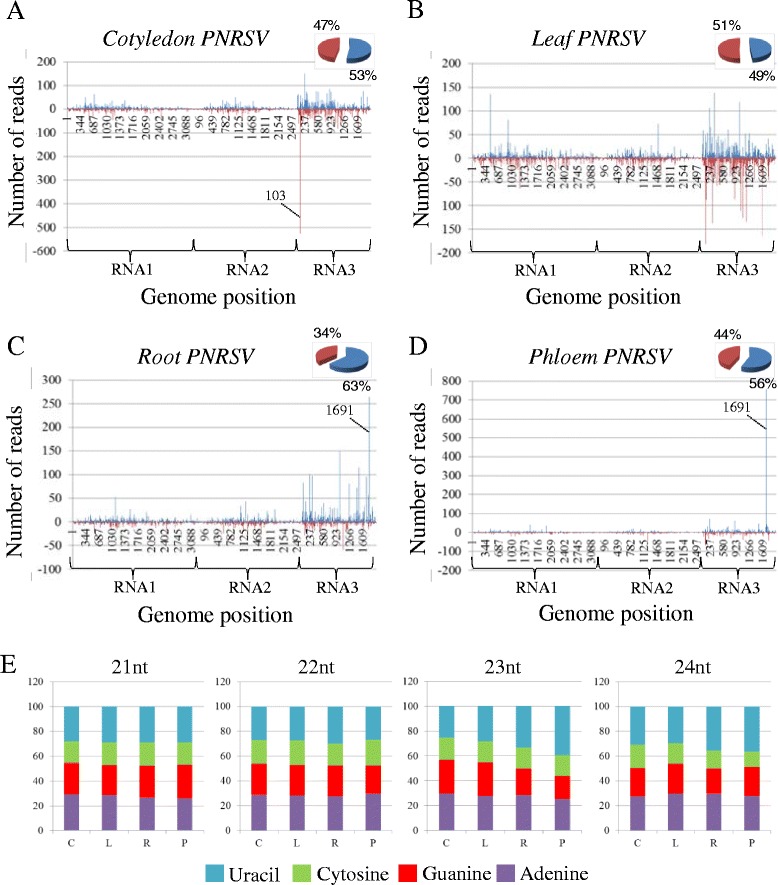
**Distribution of vsRNAs along the** ***Prunus necrotic ringspot virus*** **(PNRSV) genome in both positive and negative polarity and relative abundance of the four different 5′terminal nucleotides in the most representative species of vsRNAs. A**, **B**, **C** and **D** represent the distribution of vsRNAs along the PNRSV genome in cotyledon, leaf, root and phloem respectively, in both polarities; positive in blue and negative in red. Along abscissa axis, the three PNRSV genomic RNAs are represented. Broken lines indicate the hot-spots validated by stem-loop RT-PCR. Circular graphs (upper right corners in **A**, **B**, **C** and **D**) show the percentage of vsRNAs in each polarity. **(E)** Percentage of each 5′terminal nucleotides in all the tissues and for the most abundant vsRNAs species (21 to 24 nt).

To analyze the distribution of the vsRNAs along the virus genome, the 5′ends of the vsRNAs (16- to 35-nt) from each library were plotted against the corresponding virus sequence considering both polarity and number of reads. As it is shown in Figures 5 and 6 vsRNAs are distributed along the whole corresponding genome in all the tissues. Similar amounts of sense and antisense vsRNAs were present in cotyledon, leaf and phloem in both systems. Interestingly, in MNSV- and PNRSV-infected roots, the percentage of positive species was higher (approximately 60%) than negative ones (approximately 40%) (Figures 5C and 6C).

In *A. thaliana,* the 5′ terminal nucleotide of the sRNAs influences their selective loading into specific AGOs. Thus, investigating the 5′-terminal nucleotide of vsRNAs can give insight into vsRNA function. Figures 5E and 6E shows the percentage of vsRNAs of 21 to 24 nt with 5′ terminal Uracil, Cytosine, Guanine, or Adenine in the different tissues. We found a similar percentage for each nucleotide independently of the size, tissue and plant-virus system (Figures 5E and 6E). This result suggests the involvement of different Ago complexes in all analyzed tissues, including phloem.

### Presence of selected vsRNAs “hot-spots”was validated by stem-loop RT-PCR

As described above, both MNSV and PNRSV vsRNAs mapped along the whole corresponding virus genome. However, PNRSV vsRNA distribution was more heterogeneous with a higher accumulation along the RNA 3 (Figure 6). Additionally and in both systems, vsRNAs accumulated specifically at discrete sites along the virus genome. Three hot-spots of vsRNA accumulation located at the N-terminal half of the p42 MNSV ORF were especially prevalent. Two of them were of sense polarity and mainly composed of a unique 21-nt length vsRNA. The third MNSV hot-spot was of antisense polarity and composed of vsRNAs of 21- and 22-nt. According to their 5′ nucleotide position on the genome, these hot-spots were named as vsRNA 3078, 3099 and 3160, respectively. The relative abundance of vsRNA 3078 and 3160 was comparable in all the tissues (0.1-0.12% for vsRNA 3078 and 0.27-0.35% for vsRNA 3160) except in phloem sap were both, vsRNA 3078 and 3160, were selectively enriched becoming the most abundant vsRNAs (0.6% and 0.59% respectively). For vsRNA 3099, the highest relative abundance was observed in cotyledon (0.37%) and root (0.32%) decreasing in phloem (0.25%) and leaf (0.1%) (Figure 5). For PNRSV, two noteworthy hot-spots were observed, one antisense vsRNA of 21-nt at the 3′ UTR region of the RNA3 (vsRNA 103) which was mainly abundant in cotyledon (2.3%) and an interesting sense vsRNA of 25-nt located at the C-terminus of the coat protein (vsRNA 1691) with a high number of reads in phloem (7.3%) (Figure 6).

**Figure 7:**
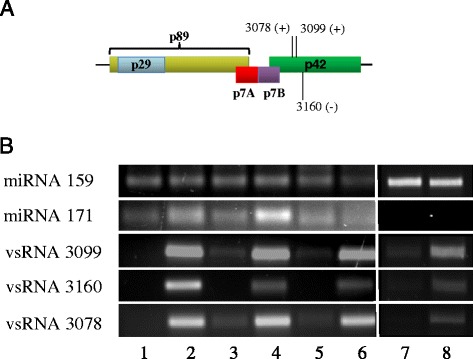
**Validation of MNSV hot-spots by stem-loop RT-PCR. (A)** Schematic representation of the complete genome of MNSV with the different open reading frames indicated by colors. Numbers represent the 5′position on the genome of the three selected hot-spots and their polarity is shown between brackets. **(B)** Stem-loop RT-PCR analysis of the three hot-spots in all the tissues. Healthy leaf (1), MNSV infected leaf (2), healthy cotyledon (3), MNSV infected cotyledon, healthy root (5), MNSV infected root (6), healthy phloem (7) and MNSV infected phloem (8). As a positive and negative controls in phloem, miR159 and miR171 respectively were used.

**Figure 8:**
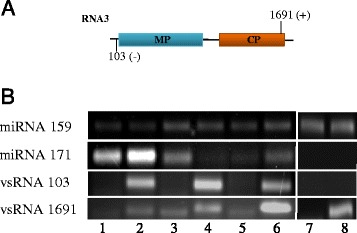
**Validation of PNRSV hot-spots by stem-loop RT-PCR. (A)** Schematic representation of the RNA3 of PNRSV. Numbers represent the 5′position on the genome of the two selected hot-spots and their polarity between brackets. **(B)** Stem-loop RT-PCR analysis of the three hot-spots in all the tissues. Healthy leaf (1), PNRSV infected leaf (2), healthy cotyledon (3), PNRSV infected cotyledon (4), healthy root (5), PNRSV infected root (6), healthy phloem (7) and PNRSV infected phloem (8). As positive and negative controls in phloem, miR159 and miR171 respectively, were used.

As these “hot-spots” may have biological relevance, we validated their presence by stem-loop RT-PCR [53]. As positive control for all tissues the conservative miR159, which is known to be also present in the phloem, was used. In addition, miR171, which has neither been observed in phloem sap by deep sequencing nor sRNA array experiments [22], was selected as a negative control to test phloem sap pureness. Figures 7 and 8 show the presence of all of the viral hot-spots and miR159 in all the tissues validating the presence of these sRNAs in our deep-sequencing data. As expected, miR171 was detected in leaf, cotyledon and root tissues but was absent in phloem. This indicates that the phloem exudates used here were pure and not contaminated with small RNAs from surrounding stem tissue.

## Discussion

RNA silencing controls countless biological processes including, in the case of plants, defence against biotic and abiotic stresses. When a plant is infected by a virus, the silencing machinery has to combine efforts to guarantee the correct development of the plant on the one hand and an appropriate defence against the pathogen on the other. This involves, among other aspects, a silencing signal with nucleotide-sequence specificity that spreads over long distances through the phloem tissue. Next generation sequencing has allowed a considerable progress in identifying and characterizing the hallmark molecules in this process, the vsRNAs. However, in most of these studies, the characterization of the vsRNAs has been usually restricted to a particular tissue. In addition, when phloem tissue was analyzed, its vsRNA population has not been compared, with very few exceptions, with sink and/or source tissues. In this work we deep-sequenced the sRNA population of four tissues; cotyledon as source, symptomatic-primary leaf and root as sink tissues and phloem as conductive tissue. In addition, we compared two different plant-virus systems, MNSV and PNRSV, infecting melon and cucumber plants, respectively.

Sequence analysis of the data revealed a remarkable difference between the total number of vsRNA reads in both systems. Consistent with our data, Donaire et al. [7] found high variability in vsRNA reads when comparing a variety of plant-virus interactions. The authors attributed this variability to differences in virus accumulation and replication, efficiency of the RNA machinery and to the mode of action of the viral silencing suppressor (VSR). Similar to that reported here, the same percentage of vsRNAs (around 57%) was obtained in MNSV infected cotyledons from melon plants. Curiously, the profile of vsRNAs of the *Cucumber mosaic virus* (CMV), a member belonging to the same family than PNRSV, showed also a lower percentage of virus-derived sRNAs (around 14%) in *Arabidopsis thaliana*.

Our study did not reveal a significant correlation between virus accumulation and number of vsRNAs. Thus, other factors like differences in virus counter defense strategies in the respective host plants likely accounts for the differences in vsRNA accumulation in the plant-virus systems tested. Little is known about the MNSV and PNRSV VSRs. For MNSV, the two viral proteins, p42 and p7B have been described to have VSR activity [50]. They were classified as weak because they delayed but did not prevent PTGS in transient expression experiments on GFP-transgenic plants. The high number of MNSV-derived sRNAs indicated that MNSV RNAs serve as highly effective substrates for DCL enzymes. The fact that large amounts of vsRNAs coexist with high titers of MNSV genome accumulation indicates that MNSV VSRs could act downstream of sRNA production in the PTGS pathway. One possibility could be that the MNSV VSRs avoid RISC assembly through vsRNA sequestering. Alternatively, MNSV replication and spreading could occur at rates that outcompete the capacity of the plant silencing machinery for its degradation. Further experiments to determine how these two proteins work at the molecular level and whether or not they interfere with the silencing signal spread are necessary. For PNRSV, no viral protein acting as a VSR has been described so far. Our finding that only very low levels of PNRSV sRNAs accumulate upon infection indicates that this virus encodes a VSR acting upstream of vsRNA production or uses another non-conventional mechanism allowing the virus to escape silencing. Examples of such mechanisms have been previously described; *Cauliflower mosaic virus* (CMV), for instance, uses decoy RNAs to evade silencing [54], *Red clover mosaic virus* (RCNMV) suppresses RNA silencing recruiting DCL1 proteins or their homologs to the replication complexes [55] and highly structured viral RNAs of human adenovirus sequester Dicer and suppress antiviral defence [56]. Like PNRSV, *Magnaporthe oryzae virus* 2 (MoV2), a dsRNA mycovirus, accumulates very low amounts of vsRNAs (0.5%). None of the viral proteins exhibit RNA silencing suppressor activity but MoV2 seems to evade silencing by replicating within viral particles [57]. Similarly, the PNRSV genome might be protected from degradation through interaction with proteins or early encapsidation. Interestingly, PNRSV, like other ilarviruses and *Alfalfa mosaic virus* (AMV), require a specific and efficient interaction of their CPs with the viral genome for replication and translation [58]. It would be interesting to know if the low accumulation level of vsRNAs observed from PNRSV is a general rule for the rest of ilarviruses and AMV.

Importantly, despite the fact that PNRSV infection hardly produced any vsRNAs, total sRNA profiles MNSV and PNRSV infected plants showed a clear increase of the 21-nt species in all the tissues being less evident in phloem. A sharp decline of 24-nt sRNA relative abundance was also observed in all the tissues except for the phloem sap where the amount remains similar and even higher than the 21-nt species. This indicates that virus infection modulates dicer activities independent of whether the viral RNA is targeted by silencing or not.

Similar to our findings, a 2.5-3 fold reduction of 24-nt sRNAs relative abundance compared to healthy plants was detected in *Citrus tristeza virus* (CTV)-infected Mexican lime and sweet orange but not in sour orange [59]. Interestingly, in the two former citrus species, CTV invades adjacent phloem-associated cells at distal regions of the plant producing small infection clusters. In less-susceptible sour orange species, the virus is able to exit sieve elements but cannot spread to adjacent cells resulting in isolated single infected cells and less than 1/10 of Mexican lime virus concentration [60]. A similar effect on 24-nt sRNAs concentration occurs in the synergistic sweet potato virus disease (SPVD) [61]. In SPVD, a phloem-limited crinivirus (*Sweet potato chlorotic stunt virus*, SPCSV) increases 600-fold the titer of an unrelated potyvirus (*Sweet potato feathery mottle virus*, SPFMV) in non-phloem tissue resulting in highly symptomatic plants. It was hypothesized that SPCSV interferes with the systemic phloem dependent signaling required for SPFMV resistance [62]. Only mobile 24-nt sRNAs have been associated with RNA-dependent DNA methylation (RdDM) in recipient cells that may trigger transcriptional gene silencing (TGS) [63]. Whether local reduction of the 24-nt abundance allows high titers of viral accumulation by affecting fundamental mechanisms related with gene expression of viral resistance in distal parts needs to be clarified.

From our results and previous studies, it appears that all size classes of sRNA are mobile. However, when we compared libraries from healthy and infected samples, the most remarkable new observation was that whereas for the rest of the tissues the ratio between the 21/24-nt sRNAs increases, it is maintained in phloem (Additional file 5: Figure S3). In infected samples, both types of sRNAs have to be loaded into sieve tubes against a concentration gradient suggesting the existence of a mechanism that actively selects the sRNAs to be transported. Analysis of the sRNA composition in phloem has been previously carried out in different systems [21,23,32] but only Yoo et al. [21] identified the small RNA species in phloem of *Cucumber yellows closterovirus* (CuYV) infected pumpkin plants. In contrast to what we report here, the authors observed a distribution pattern clearly different from the healthy one. However, this apparent contradiction needs to be carefully considered since CuYV vsRNAs, mostly of 20 and 21-nt, represented 57% of total sRNAs. In our study, due to the low percentage of vsRNAs in infected phloem (10% in melon and close to 0% in cucumber), the bulk of the sRNA population in phloem belongs to the endogenous sRNA group. Consequently the size distribution analysis of the host genome sRNAs showed the same pattern than for the total sRNAs (Additional file 8 Figure S6 and Additional file 9: Figure S7). To better understand host responses to infection and possibly to gain insight into transported host sRNAs it will be interesting to see which host sRNAs exhibit modulated abundance in the different tissues upon infection. Additionally, the fact that despite an overall increase of 21-nt sRNAs and decrease of 24-nt sRNAs upon infection in leaves, cotyledons and roots the ratio between 21-nt to 24-nt sRNAs remains constant in phloem tissue could suggest that 24-nt sRNAs are the main transported sRNA species in these plants. In addition, as the percentage of 24-nt species in phloem is similar upon infection (Additional file 8: Figure S6 and Additional file 9: Figure S7), a qualitative change of these small molecules could alert the distal parts of the plant. Some lines of evidence have indicated a role for sRNAs with different sizes in long-distance transmission of RNA silencing [27-29]. Alternatively, viruses may have found a way to selectively inhibit transport of 21-nt sRNA species.

The antiviral silencing machinery in Arabidopsis acts mainly through DCL4, which produces 21-nt small RNAs [12]. As mentioned above, and consistently with this, vsRNAs in melon and cucumber plants were mainly 21-nt in size, which correlated with a clear increase in the expression level of the DCL4 transcript in all the tissues, and indicates that dicer functions and dicing patterns are conserved between species. Accumulation of the 21-nt long vsRNAs in both virus/plant systems indicates that neither of the two unrelated viruses used in this study impaired the DCL4 function. Thus DCL4 seemed to be the most important antiviral enzyme in this plant genus. In all tissues, our results also indicated that whereas MNSV vsRNAs derived equally from whole genomic RNA, PNRSV vsRNAs mapped at a higher percentage to RNA3. It is noteworthy that all the tissues in both systems showed equivalent amounts of sense and antisense vsRNAs, except for roots, where the number of vsRNAs with a positive polarity increased. The bias to the genomic sense strand in roots could support a model by which the secondary structures within viral single strand RNA contribute to vsRNA generation. But why does this happen only in roots? A previous work by our group revealed high levels of MNSV accumulation in infected melon roots [64]. This differential tropism was also observed recently for citrus species infected with Citrus tristeza virus (CTV) [65]. In MNSV-infected plants, the concentration of dsRNAs, as a measure of replication, was 5-fold lower in roots than in cotyledons, despite the amount of virus being comparable in both tissues. This observation could consequently imply a greater accumulation of viral genomic RNA, which could serve as a substrate for DCL cleavage, and would support the observed increase in sense vsRNAs in our study. According to Gosalvez-Bernal et al. [64], one would imagine roots to be like a prelude to the virus being loaded into the internal phloem; in other words, a bottleneck where the replication of the virus would decrease, and would accumulate while waiting to enter the vascular system in order to spread. Alternatively, Andika et al. [66] provided evidence that the RNA silencing mechanism is less effective in roots than in leaves, which could explain the substantial viral RNA accumulation in this tissue. However, and as stated above, the accumulation levels of vsRNAs in MNSV-infected roots were similar to the other tissues, while other mechanisms, which differed from RNA silencing, might account for this differential tissue tropism. Further experiments that investigate this increase in sense vsRNAs in MNSV- and PNRSV-infected roots, as well as the molecular mechanism that underlies reduced viral replication in this tissue, will provide new insights into this phenomenon. According to previous work, sRNA loading into specific AGO complexes is conditioned by the 5′ terminal nucleotide [67-69]. In the present work, none of the vsRNA 5′ends showed any prevalence. High variability in this aspect [36,70] further evidences the great complexity of this mechanism and the wide range of AGO complexes, presumably with different roles, which might be involved in the process.

Finally, our comparative sRNA profiling analysis has revealed that MNSV and PNRSV infections both lead to the production of mainly 21-nt vsRNAs, which is consistent with DCL4 being the main antiviral silencing component in plants. The fact that PNRSV infection hardly led to the production of vsRNAs indicates that this virus has evolved mechanisms to avoid being targeted by the host silencing machinery. However, both viruses strongly altered the total sRNA profile, which reveals that virus infection modulates the host gene expression. In future analyses, it will be interesting to see whether these changes are host defense responses or whether the virus actively uses silencing to down-regulate defense. The different pattern of sRNAs found in phloem tissue is probably one of the most interesting observations to have emerged from our comprehensive sRNA analysis. Given that viral or host sRNAs are transported over long distances, it will be interesting to analyze the identity of the sRNA population in phloem compared to other tissues, and to also analyze the target gene expression in sink tissues.

## Conclusions

We have compared the sRNA profile of four different tissues, including source, sink and conductive (phloem) tissues, in two different pathosystems for the first time. Our results indicate that the antiviral silencing machinery in melon and cucumber acts mainly through DCL4. One of the most interesting results to have emerged from our sRNA analysis is that the total sRNA pattern in phloem remains unchanged upon infection, unlike the other analyzed tissues. In addition, and independently of the accumulation level of vsRNAs, both viruses were able to modulate the host sRNA pattern.

## Methods

### Plant material and RNA extraction

Cotyledons of both *Cucumis melo* cv. Galia and *Cucumis sativus* cv. Supermarketer plants were inoculated with purified virions and crude virus-containing extracts from infected plants respectively, 9 days after germination. As a control, mock-inoculated plants were grown in parallel and under the same greenhouse conditions (16 hours at 24°C and 8 hours at 18°C in daylight conditions). Inoculated-cotyledons, root, phloem and primary leaf from mock-inoculated and symptomatic infected plants were harvested 15 days after inoculation (15 dpi).

To collect the exudates, the first petiole and the stem internode between the first and second leaf were cut with a sterile razor blade and with the help of a pipet the phloem was collected in an Eppendorf-tube containing 1 ml of TRIzol.

Total RNA extraction was identical for all the samples and carried out by using conventional TRIzol (Sigma-Aldrich) protocol according to the manufacturer’s instructions. The quality of the samples was verified by Northern-blot analysis using specific digoxigenin-labelled riboprobes for each virus and evaluating both A260/230 and A260/280 absorption ratios (Nanodrop- Thermo Scientific).

### Small RNA PEG fractionation

To enrich the samples in sRNAs, 3 volumes of polyethylene glycol (MW 8000) and NaCl to final concentrations of 5% and 500 mM respectively were added. After incubation on ice for 30 minutes, the samples were centrifuged at 15000 rpm for 15 min at 4°C. The upper phase was transferred to a new tube and 4 volumes of phenol were added. Then the tubes were vortexed thoroughly and centrifuged during 2 minutes at maximum speed. Afterwards the upper phase was precipitated and the pellet containing sRNAs was resuspended in 50 μl of water.

### Silver staining

1 μg of each fractionated sample was separated in a 17% polyacrylamide/7 M urea gel for the subsequent silver staining. For this purpose, the gel was soaked for 45 minutes in ethanol/H_2_O/acetic acid (ratio 50:40:1) followed by a second 45 minutes incubation in the same solution with a ratio of 10:89:1. Thereafter the gel was dipped in 150 ml of H_2_O with 0.3 g AgNO_3_ for 45 minutes. Then, the gel was rinsed 3 times with sterile water and developed in a formaldehyde solution (0.5 M KOH, 1.2 ml 37% formaldehyde, in 150 ml H_2_O). The reaction was stopped by washing with sterile water.

### Isotopic Northern-blot analysis

In order to detect vsRNAs, melon (2 μg) and cucumber (4 μg) fractionated RNA samples were separated in a 17% polyacrylamide/ 7 M urea gel in 0.5X TBE, transferred onto a positively-charged nylon membrane using the Bio-Rad Trans-Blot Cell and covalently UV cross-linked to the membrane (700 × 100 μJ/cm^2^). Detection of the vsRNAs was carried out using specific riboprobes labeled with (α-^32^P) ATP. After overnight hybridization, the membranes were washed twice with 2X SSC plus 0.1% SDS for 10 min at room temperature, and once with 0.1X SSC plus 0.1% SDS at 55°C for 15 min, and examined with a bioimage analyzer (Fujifilm FLA-5100).

### sRNA sequence processing

Production and sequencing of the libraries were carried out by the biotechnology company GenoScreen (http://www.genoscreen.com). Small RNAs were fractionated from total RNA by acrylamide gel purification. Single strand ligation of 3′ and 5′ adaptors was done before a second acrylamide gel purification. Reverse transcription and PCR amplification were performed to generate the cDNA colonies template library. To verify quality and ensure that there was no contamination, libraries were then titrated by a 1x50bp run on HiSeq2000.

A total of 16 sRNA libraries were sequenced on an Illumina genomic DNA analyzer in 1 HiSeq 2000® channel. Adapter trimming and cleaning of the reads was carried out by the same company. Most of the inserts found had a length ranging from 18 to 26 bases.

Each sRNA library was aligned with the corresponding plant (Melonomics: melon_genome_pseudomolecules_V3.5.fasta http://www.melonomics.net Cucumber genome database: ACHR00000000.1 http://www.cucumber.genomics.org.cn) and known virus genomes (MNSV Al: DQ339157 or PNRSV NcM1.NctSp.mur1: AJ306818) by the Bioinformatics Service at the IBMCP (http://www.ibmcp.upv.es). Count of matching sequence reads were normalized to the total number of reads after last filtering step and given in parts per million

### Hot-spot validation by Stem-loop RT-PCR

We used the protocol described by Varkonyi-Gasic, et al. [53] with some modifications. For the pulse reverse transcription reaction we mixed 0.5 μM of appropriate stem-loop RT primer (Additional file 10: Table S3), 1 mM dNTPs and 100 ng of the corresponding RNA in a volume of 14.9 μl and incubated at 65°C during 5 minutes and then on ice. Then, 200 U of RevertAid Reverse Transcriptase (Thermo Scientific; http://www.thermoscientific.com/en/home.html) and 40 U RNase Inhibitor were added in a final volume of 20 μl and pulsed RT was performed as follow: 30 min at 16°C, pulsed RT of 60 cycles at 30°C for 30 s, 42°C for 30 s and 50°C for 1 s. Then samples were incubated at 85°C for 5 min. PCR was carried out with the following components: 0.2 μM of each primer (Additional file 10: Table S3), 0.2% PVP, 0.2 mM dNTP’s, 0.5 mM MgCl2 and 2 U GoTaq (Promega; http://www.promega.es). The PCR reaction was performed as follows: 2 min at 94°C, 25 cycles 15 s at 94°C and 1 min at 60°C.

### Identification of AtDCL1-4 genes homologs in both Cucumis sativus and Cucumis melo genomes

*Arabidopsis thaliana* DCL proteins (DCL1; At1g01040, DCL2; At3g03300, DCL3; At3g43920 and DCL4; At5g 20320) were blasted against the corresponding database (http://www.phytozome.net and http://www.melonomics.net) to identify the four homologues in *C. sativus* and *C. melo.* Accession numbers: Cucsa.260100, Cucsa.356600, Cucsa.055950, Cucsa.350750 (*C. sativus) and* MELO3C005929P1/30P1, MELO3C010042P1, MELO3C011495P1, MELO3C010254P1 (*C. melo).*

### Real-time qRT-PCR analysis

To analyze the differential expression of DCL transcripts, qRT-PCR was performed with 100 ng of total RNA using one step SYBR PrimeScript RT-PCR Kit II (Takara) following the manufacturer’s recommendations in an Applied Biosystems 7500 Real-Time PCR System. Each biological replicate was assayed in triplicate. Gene-specific oligonucleotide primers were designed using Primer Express® version 3.0 software (Applied Biosystems). Primer information is shown in Additional file 11: Table S4. Expression levels for target genes were normalized to Elongation Factor 1-alpha (EF1 α) and phosphatase 2A regulatory subunit (PP2A) and fold expression changes compared to the healthy controls calculated using the ΔΔthreshold cycle (Ct) method.

## Additional files

Additional file 1: Table S1. Distribution of the number of reads in each sRNA library according to the insert size range. Additional file 2: Figure S1. Detection of MNSV and PNRSV by Northern-blot analysis. (A) Detection by Northern blot analysis of MSNV RNAs in melon-plant leaf, cotyledon, root and phloem inoculated with purified virions (lanes 4, 5, 6 and 8 respectively). Total RNA extracts obtained from mock inoculated plants were used as healthy controls (lanes 1, 2, 3 and 7; leaf, cotyledon, root and phloem respectively). MNSV genomic and subgenomic RNA positions are indicated in the margins. Relative sample loading is inferred from ethidium bromide staining of plant rRNA (bottom panel). (B) Detection by Northern-blot analysis of PNRSV RNAs in cucumber-plant leaf, cotyledon, root and phloem inoculated with crude virus-containing extracts from infected plants (lanes 4, 5, 6 and 8 respectively). Total RNA extracts obtained from mock inoculated plants were used as healthy controls (lanes 1, 2, 3 and 7; leaf, cotyledon, root and phloem respectively). PNRSV RNA4 and RNA3 positions are indicated in the margins. Relative sample loading is inferred from ethidium bromide staining of plant rRNA (bottom panel). Additional file 3: Figure S2. Virus titer calculation by Dot-blot analysis. Serial dilutions of equivalent amounts of total RNA (totRNA) from infected cotyledon, leaf and root were analyzed with the corresponding digoxigenin-labelled riboprobe. Additional file 4: Table S2. Virus titer in each infected tissue. Virus concentration in both PNRSV and MNSV infected tissues in arbitrary units (Java image processing program, ImageJ). Ratio between both virus loads for each tissue and dilution is also shown. Additional file 5: Figure S3. Representation of the ratio between the percentages of 21/24 nt sRNAs. (A) Ratio between the percentages of 21/24 nt sRNAs for the eight libraries of melon. (B) Ratio between the percentages of 21/24 nt sRNAs for the eight libraries of cucumber. Additional file 6: Figure S4. Expression levels of melon DCL1, DCL2, DCL3 and DCL4 genes in MNSV-infected melon plants determined by real-time qRT-PCR analysis. Values were first normalized to Elongation Factor 1-alpha (EF1 α) and phosphatase 2A regulatory subunit (PP2A) expression level and then made relative to the mRNA amount in the control, which refers to healthy plants. Three biological repetitions were carried out. Expression levels are expressed as means +/- standard errors. Additional file 7: Figure S5. Expression levels of cucumber DCL1, DCL2, DCL3 and DCL4 genes in PNRSV-infected cucumber plants determined by real-time qRT-PCR analysis. Values were first normalized to Elongation Factor 1-alpha (EF1 α) and phosphatase 2A regulatory subunit (PP2A) expression level and then made relative to the mRNA amount in the control, which refers to healthy plants. Three biological repetitions were carried out. Expression levels are expressed as means +/- standard errors. Additional file 8: Figure S6. Endogenous sRNA size distribution in healthy and MNSV infected plants. Graphic representation of the percentage of endogenous sRNAs from 16 to 35-nt in size. For each tissue the amount of sRNAs in healthy (green) and infected plants (red) is shown. Additional file 9: Figure S7. Endogenous sRNA size distribution in healthy and PNRSV infected plants. Graphic representation of the percentage of endogenous sRNAs from 16 to 35-nt in size. For each tissue the amount of sRNAs in healthy (green) and infected plants (red) is shown. Additional file 10: Table S3. Stem-loop RT-PCR primers. 5′-3′ sequence of primers used for the validation of the different hot-spots and controls. (*) Hot-spots with antisense polarity. Additional file 11: Table S4 Real-time polymerase chain reaction primers.
