# Supplementary material for: Comparative analysis among the small RNA populations of source, sink and conductive tissues in two different plant-virus pathosystems
Source: BMC Genomics. 2015 Feb 22;16(1):117. doi: 10.1186/s12864-015-1327-5 (PMC4345012; doi:10.1186/s12864-015-1327-5)
Supplement: Additional file 1: Table S1. — Distribution of the number of reads in each sRNA library according to the insert size range. [file 12864_2015_1327_MOESM1_ESM.pptx]

## Slide 1
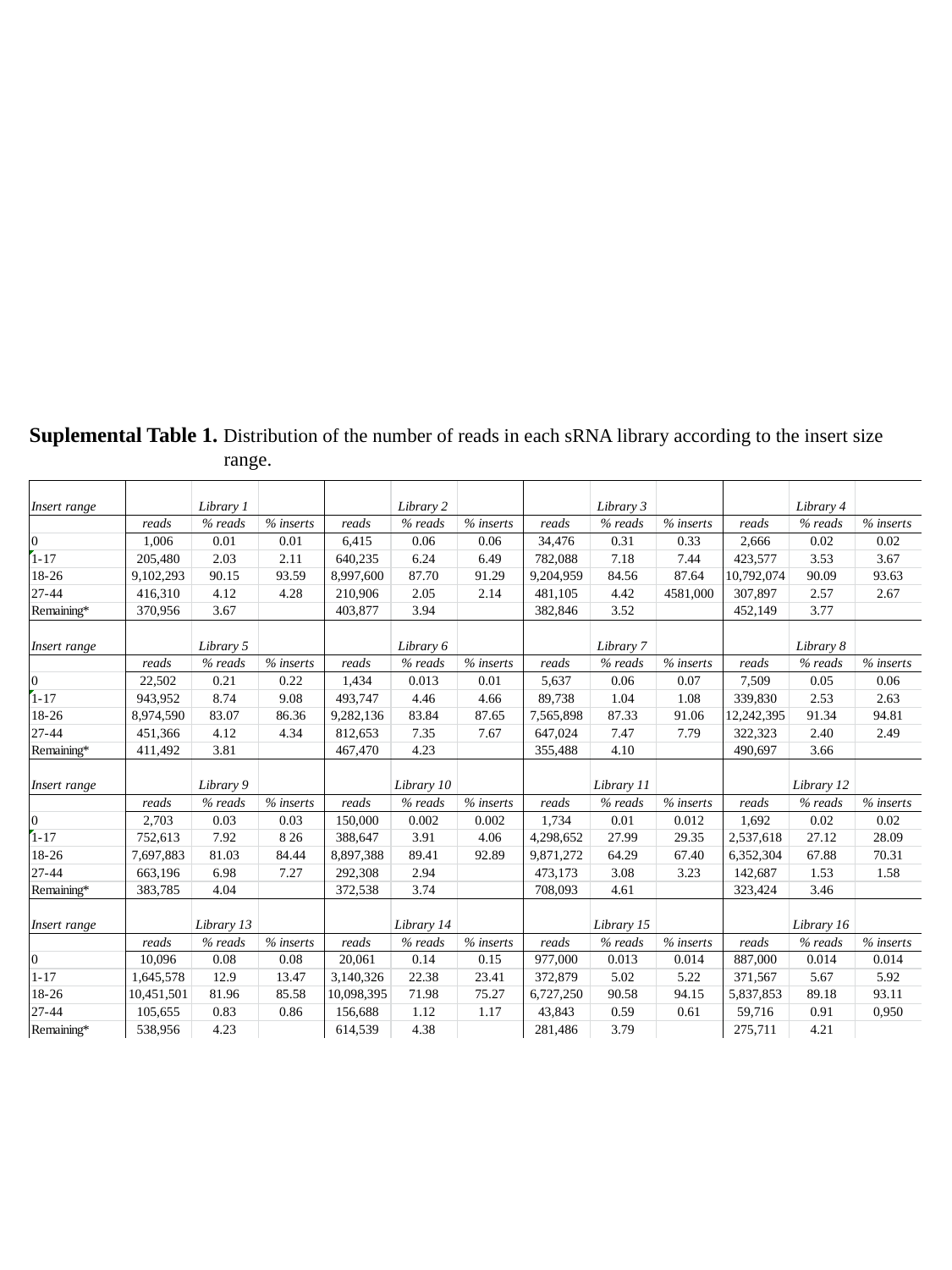

Suplemental Table 1. Distribution of the number of reads in each sRNA library according to the insert size
 range.
