# Supplementary figures and images for: Comparative analysis among the small RNA populations of source, sink and conductive tissues in two different plant-virus pathosystems
Source: BMC Genomics. 2015 Feb 22;16(1):117. doi: 10.1186/s12864-015-1327-5 (PMC4345012; doi:10.1186/s12864-015-1327-5)

## Slide 1
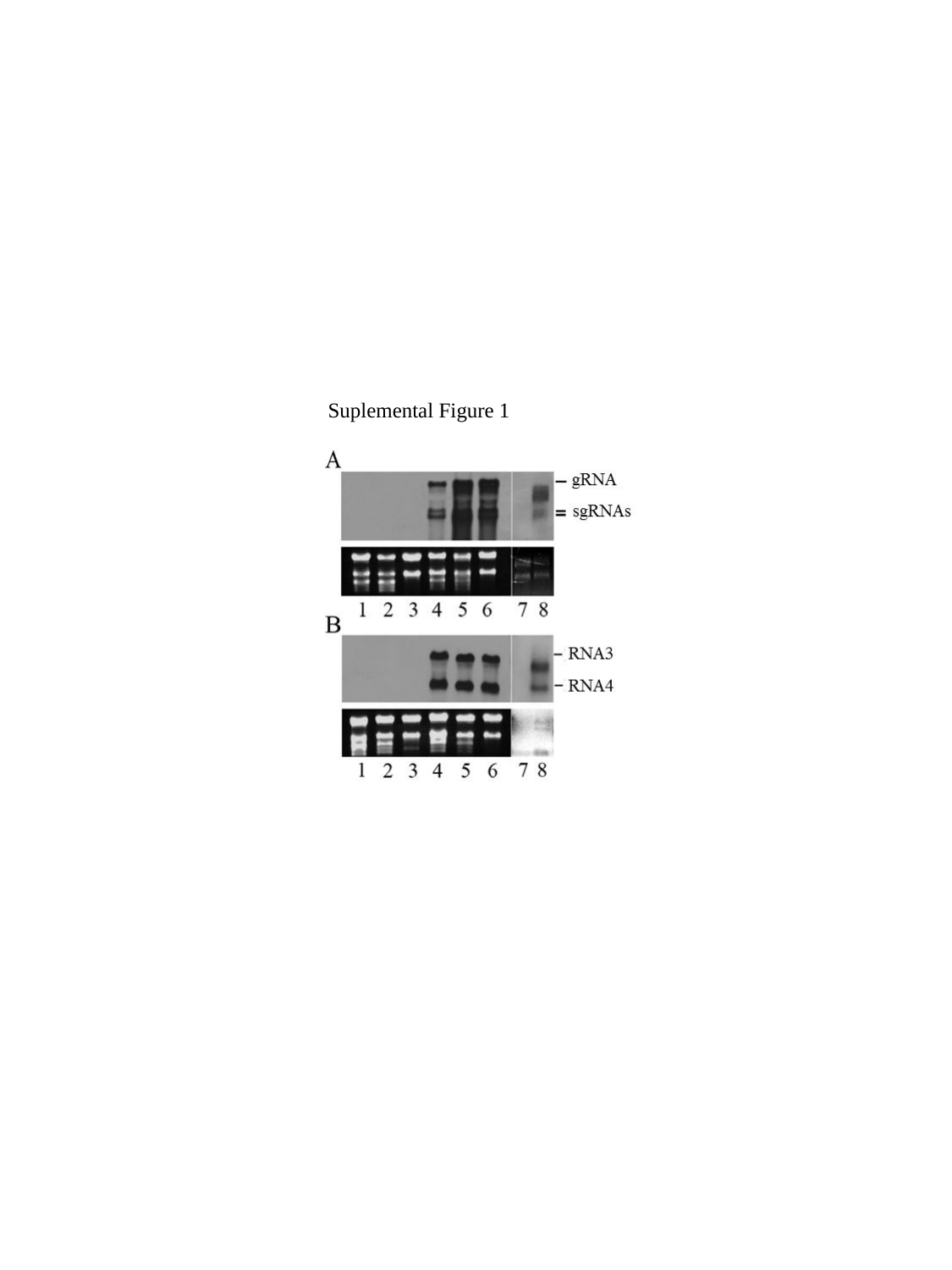

Suplemental Figure 1

Supplement: Additional file 2: Figure S1. — Detection of MNSV and PNRSV by Northern-blot analysis. (A) Detection by Northern blot analysis of MSNV RNAs in melon-plant leaf, cotyledon, root and phloem inoculated with purified virions (lanes 4, 5, 6 and 8 respectively). Total RNA extracts obtained from mock inoculated plants were used as healthy controls (lanes 1, 2, 3 and 7; leaf, cotyledon, root and phloem respectively). MNSV genomic and subgenomic RNA positions are indicated in the margins. Relative sample loading is inferred from ethidium bromide staining of plant rRNA (bottom panel). (B) Detection by Northern-blot analysis of PNRSV RNAs in cucumber-plant leaf, cotyledon, root and phloem inoculated with crude virus-containing extracts from infected plants (lanes 4, 5, 6 and 8 respectively). Total RNA extracts obtained from mock inoculated plants were used as healthy controls (lanes 1, 2, 3 and 7; leaf, cotyledon, root and phloem respectively). PNRSV RNA4 and RNA3 positions are indicated in the margins. Relative sample loading is inferred from ethidium bromide staining of plant rRNA (bottom panel). [file 12864_2015_1327_MOESM2_ESM.pptx]

## Slide 1
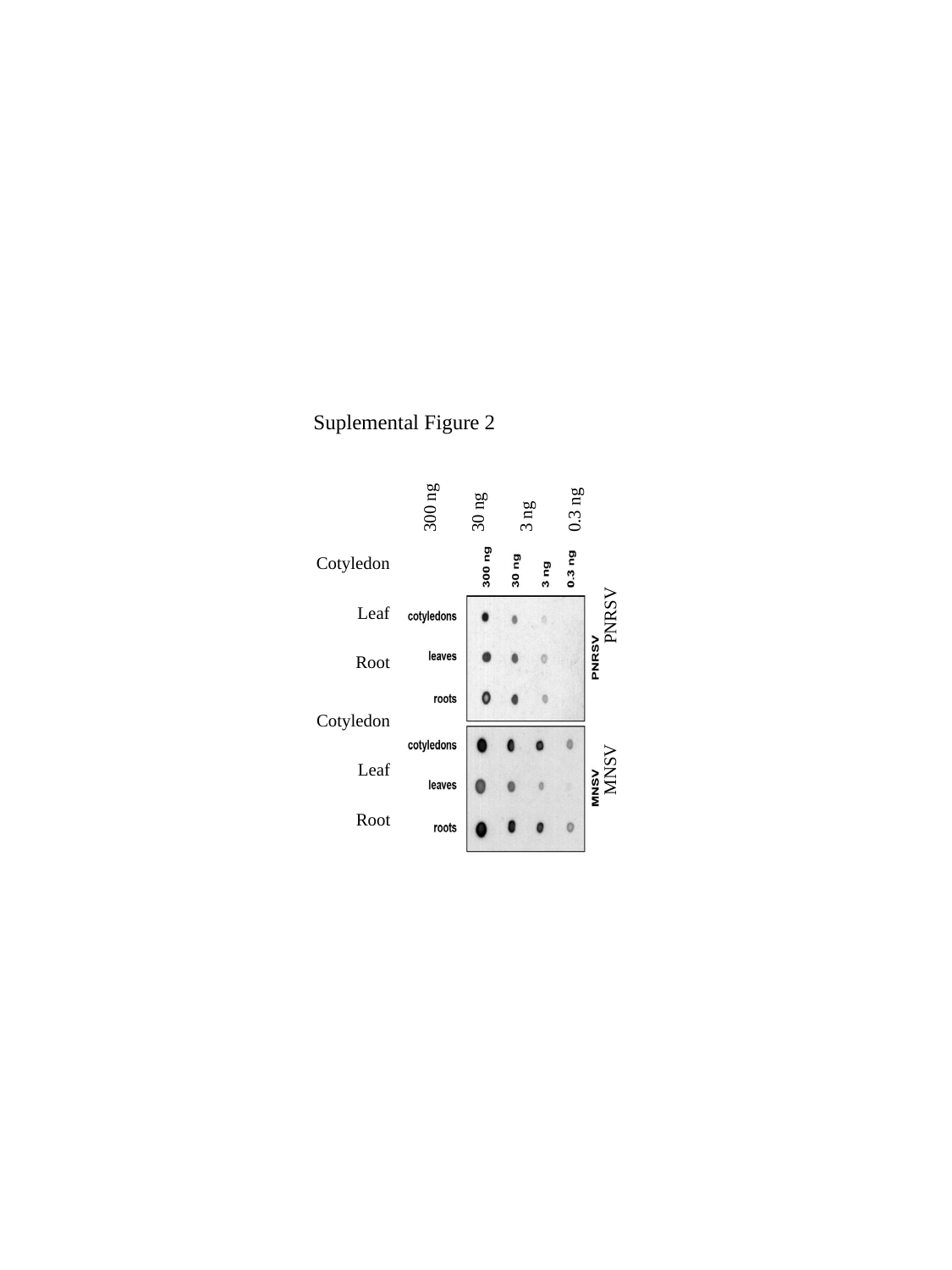

Suplemental Figure 2
300 ng
0.3 ng
30 ng
3 ng
Cotyledon
Leaf
PNRSV
Root
Cotyledon
Leaf
MNSV
Root

Supplement: Additional file 3: Figure S2. — Virus titer calculation by Dot-blot analysis. Serial dilutions of equivalent amounts of total RNA (totRNA) from infected cotyledon, leaf and root were analyzed with the corresponding digoxigenin-labelled riboprobe. [file 12864_2015_1327_MOESM3_ESM.pptx]

## Slide 1
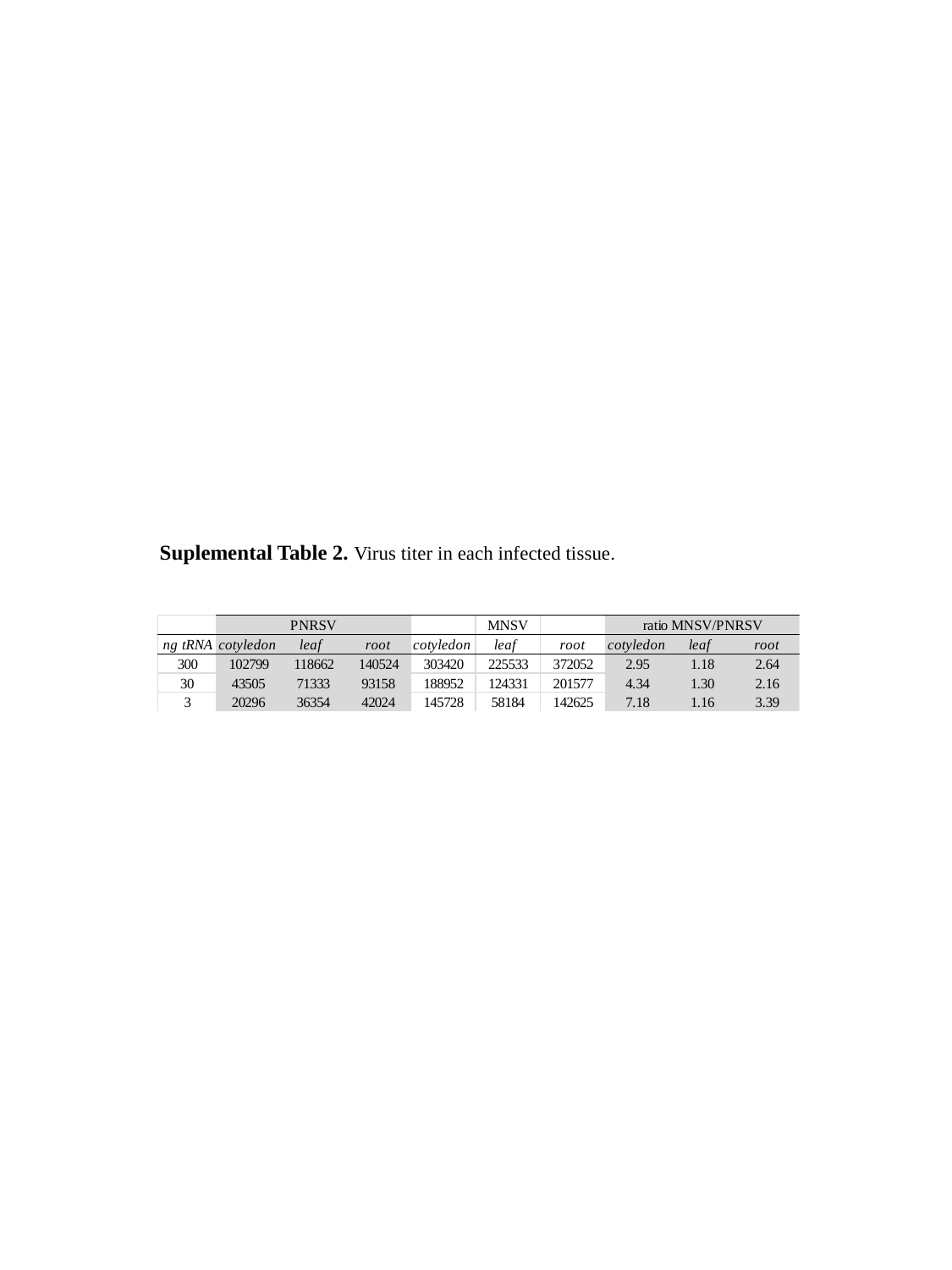

Suplemental Table 2. Virus titer in each infected tissue.

Supplement: Additional file 4: Table S2. — Virus titer in each infected tissue. Virus concentration in both PNRSV and MNSV infected tissues in arbitrary units (Java image processing program, ImageJ). Ratio between both virus loads for each tissue and dilution is also shown. [file 12864_2015_1327_MOESM4_ESM.pptx]

## Slide 1
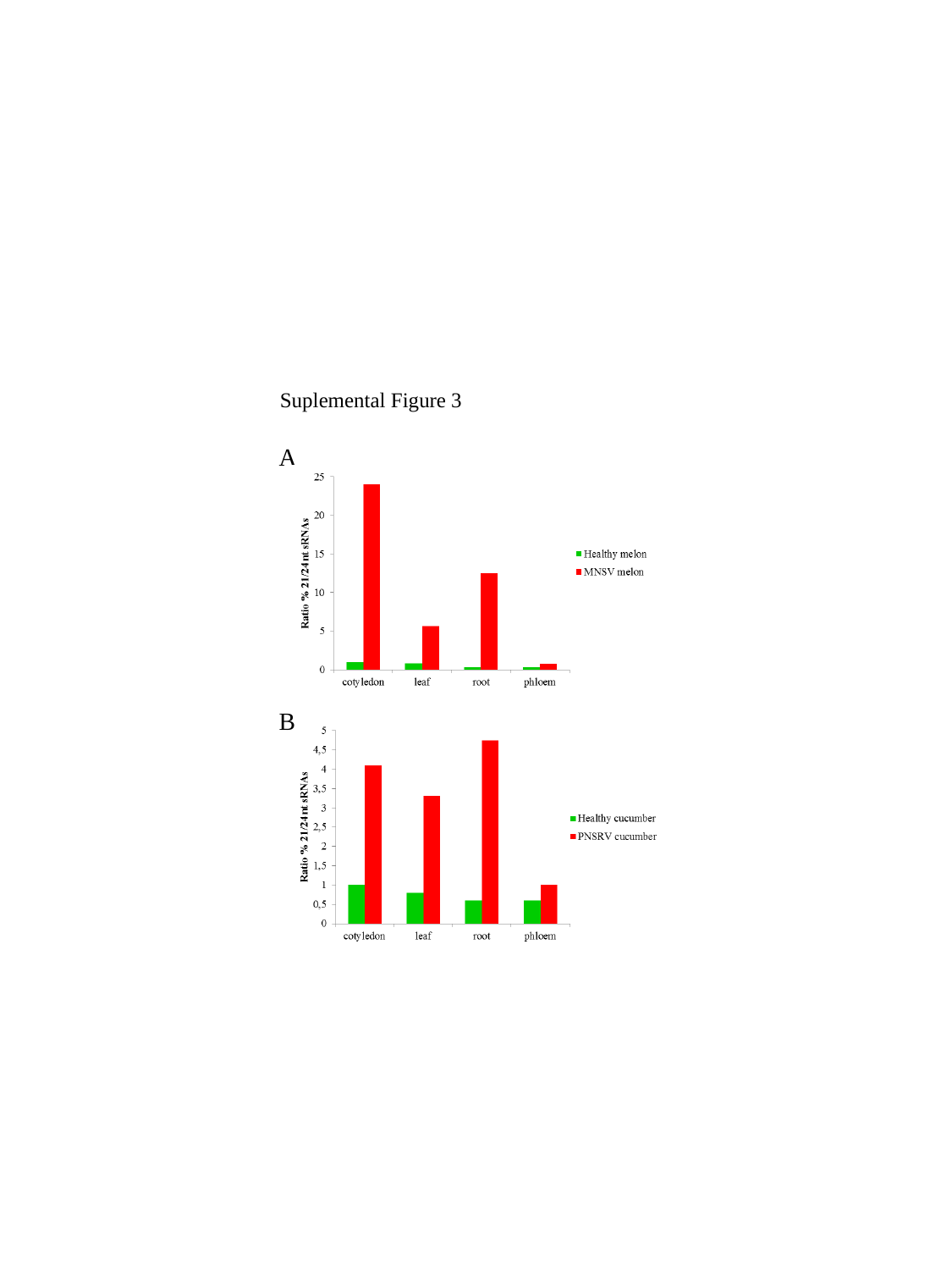

Suplemental Figure 3
A
B

Supplement: Additional file 5: Figure S3. — Representation of the ratio between the percentages of 21/24 nt sRNAs. (A) Ratio between the percentages of 21/24 nt sRNAs for the eight libraries of melon. (B) Ratio between the percentages of 21/24 nt sRNAs for the eight libraries of cucumber. [file 12864_2015_1327_MOESM5_ESM.pptx]

## Slide 1
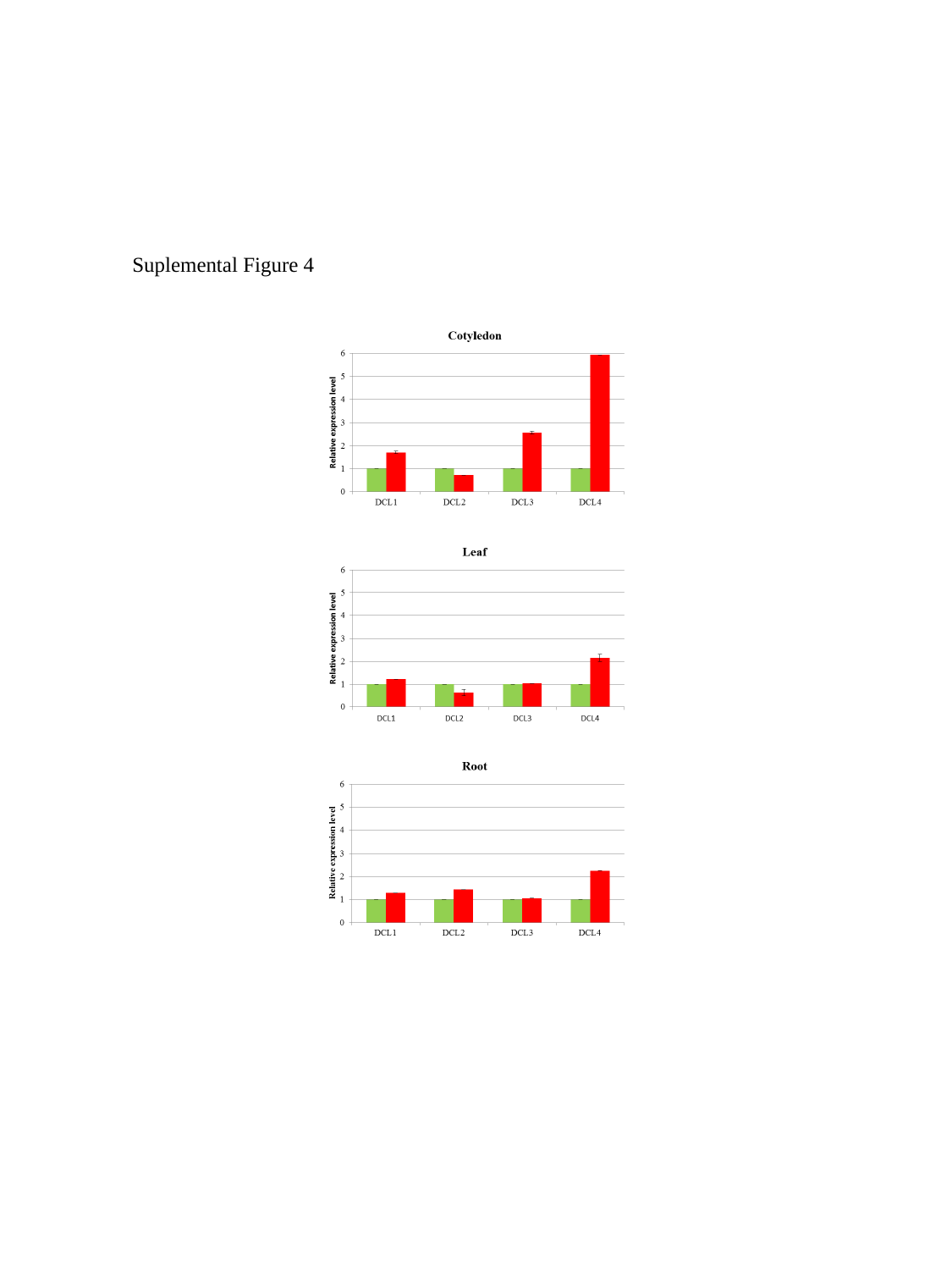

Suplemental Figure 4

Supplement: Additional file 6: Figure S4. — Expression levels of melon DCL1, DCL2, DCL3 and DCL4 genes in MNSV-infected melon plants determined by real-time qRT-PCR analysis. Values were first normalized to Elongation Factor 1-alpha (EF1 α) and phosphatase 2A regulatory subunit (PP2A) expression level and then made relative to the mRNA amount in the control, which refers to healthy plants. Three biological repetitions were carried out. Expression levels are expressed as means +/- standard errors. [file 12864_2015_1327_MOESM6_ESM.pptx]

## Slide 1
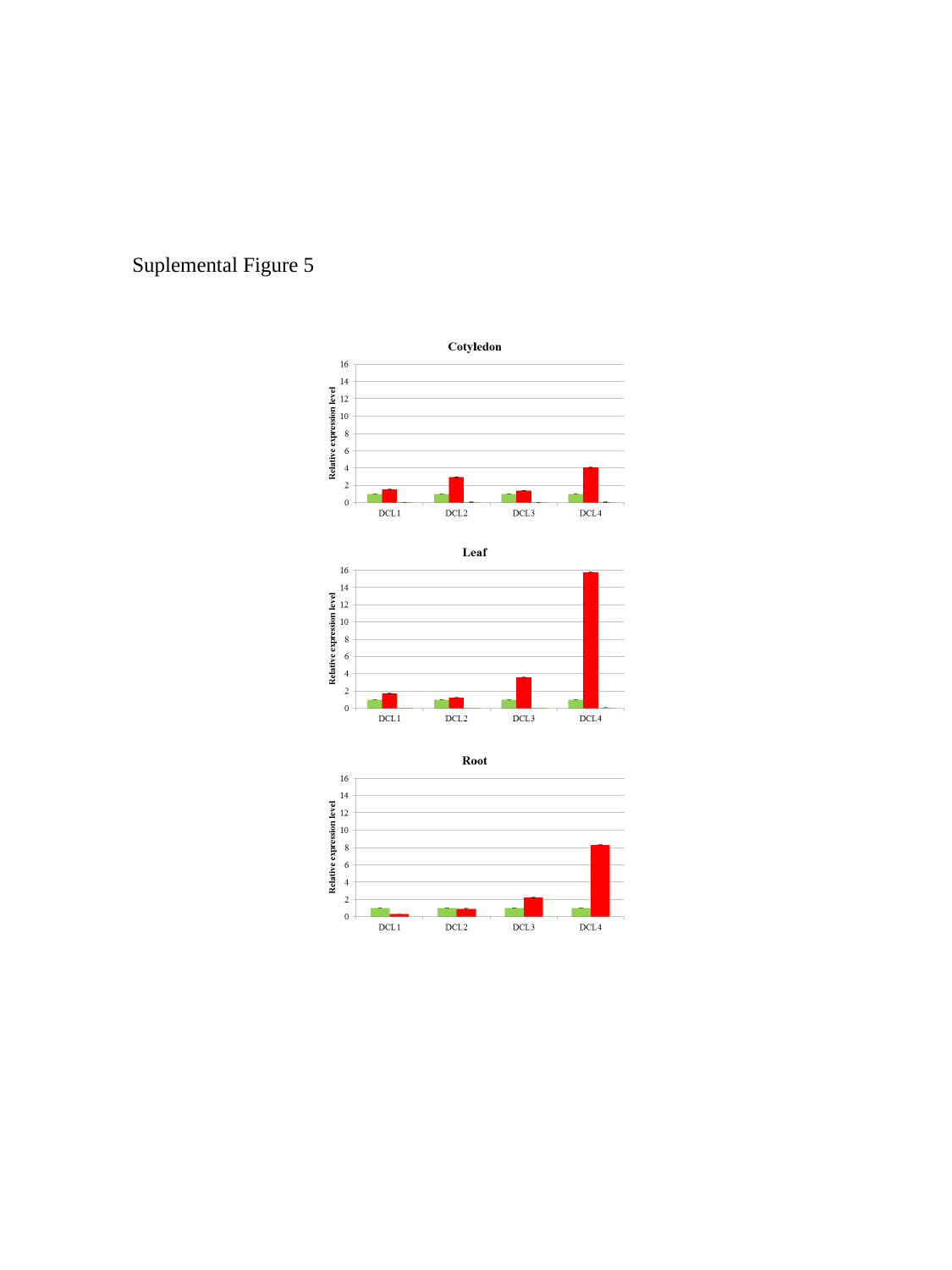

Suplemental Figure 5

Supplement: Additional file 7: Figure S5. — Expression levels of cucumber DCL1, DCL2, DCL3 and DCL4 genes in PNRSV-infected cucumber plants determined by real-time qRT-PCR analysis. Values were first normalized to Elongation Factor 1-alpha (EF1 α) and phosphatase 2A regulatory subunit (PP2A) expression level and then made relative to the mRNA amount in the control, which refers to healthy plants. Three biological repetitions were carried out. Expression levels are expressed as means +/- standard errors. [file 12864_2015_1327_MOESM7_ESM.pptx]

## Slide 1
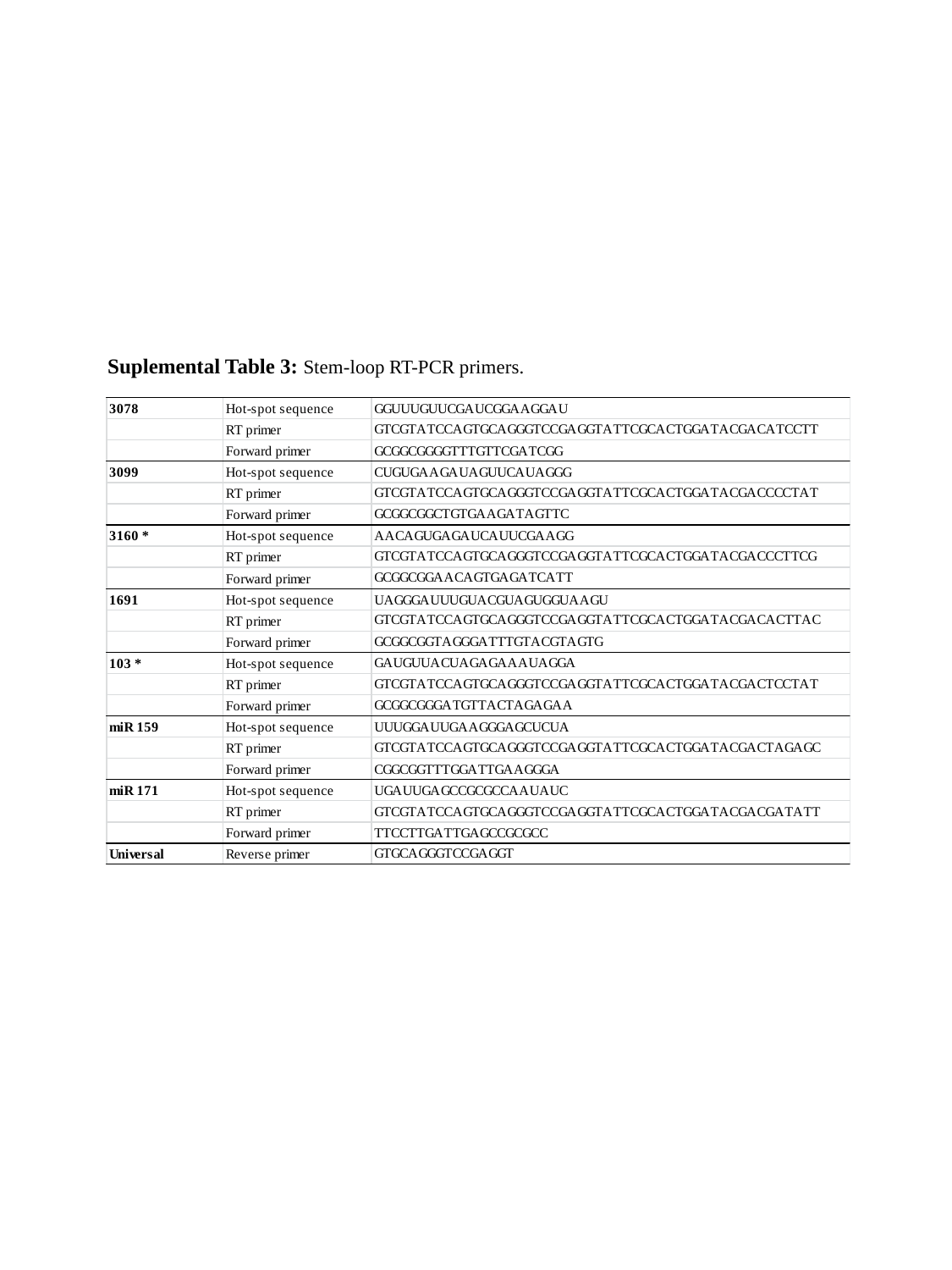

Suplemental Table 3: Stem-loop RT-PCR primers.

Supplement: Additional file 10: Table S3. — Stem-loop RT-PCR primers. 5′-3′ sequence of primers used for the validation of the different hot-spots and controls. (*) Hot-spots with antisense polarity. [file 12864_2015_1327_MOESM10_ESM.pptx]

## Slide 1
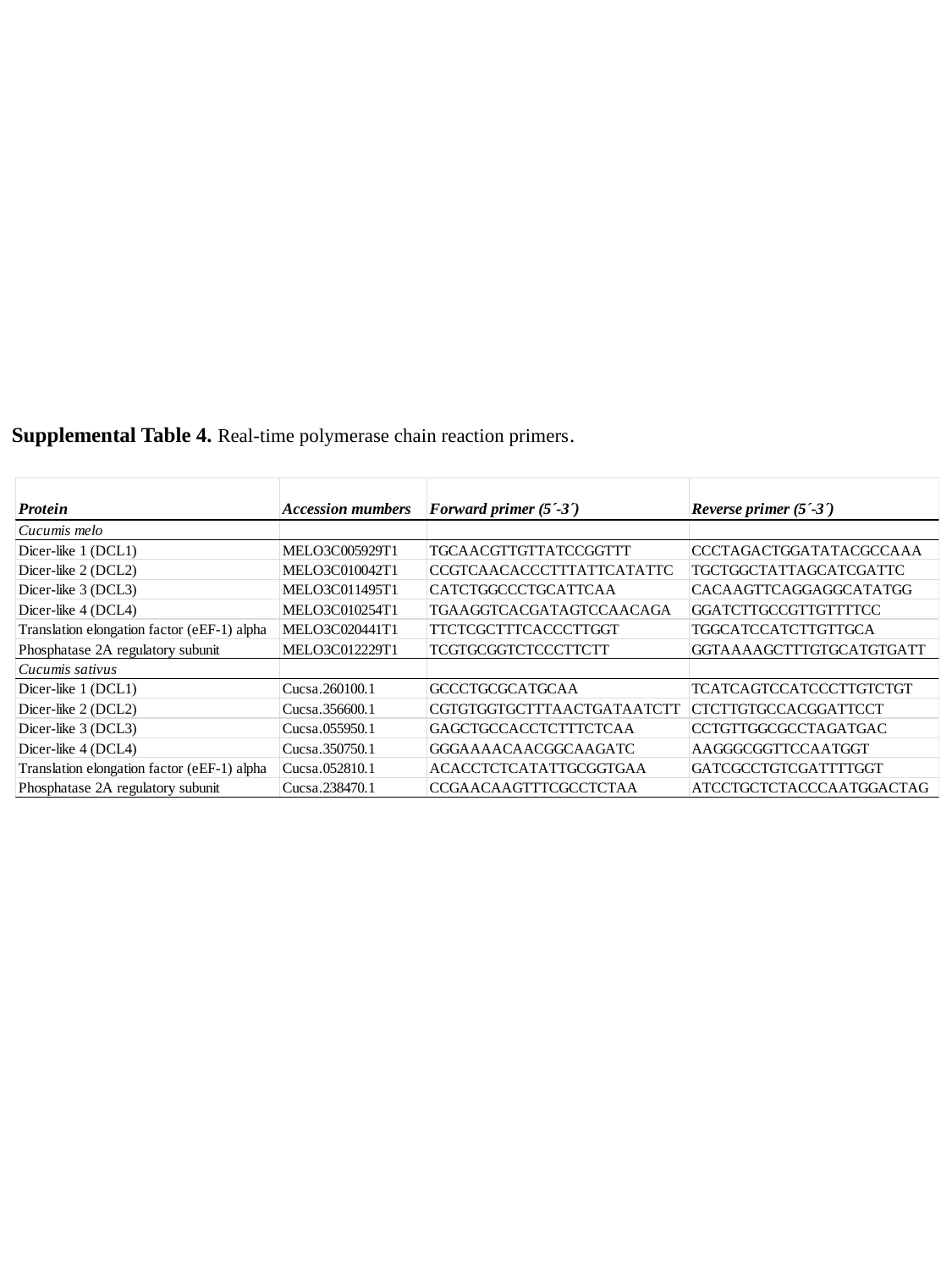

Supplemental Table 4. Real-time polymerase chain reaction primers.

Supplement: Additional file 11: Table S4 — Real-time polymerase chain reaction primers. [file 12864_2015_1327_MOESM11_ESM.pptx]
