# Supplementary material for: Comparative analysis among the small RNA populations of source, sink and conductive tissues in two different plant-virus pathosystems
Source: BMC Genomics. 2015 Feb 22;16(1):117. doi: 10.1186/s12864-015-1327-5 (PMC4345012; doi:10.1186/s12864-015-1327-5)
Supplement: Additional file 8: Figure S6. — Endogenous sRNA size distribution in healthy and MNSV infected plants. Graphic representation of the percentage of endogenous sRNAs from 16 to 35-nt in size. For each tissue the amount of sRNAs in healthy (green) and infected plants (red) is shown. [file 12864_2015_1327_MOESM8_ESM.pptx]

## Slide 1
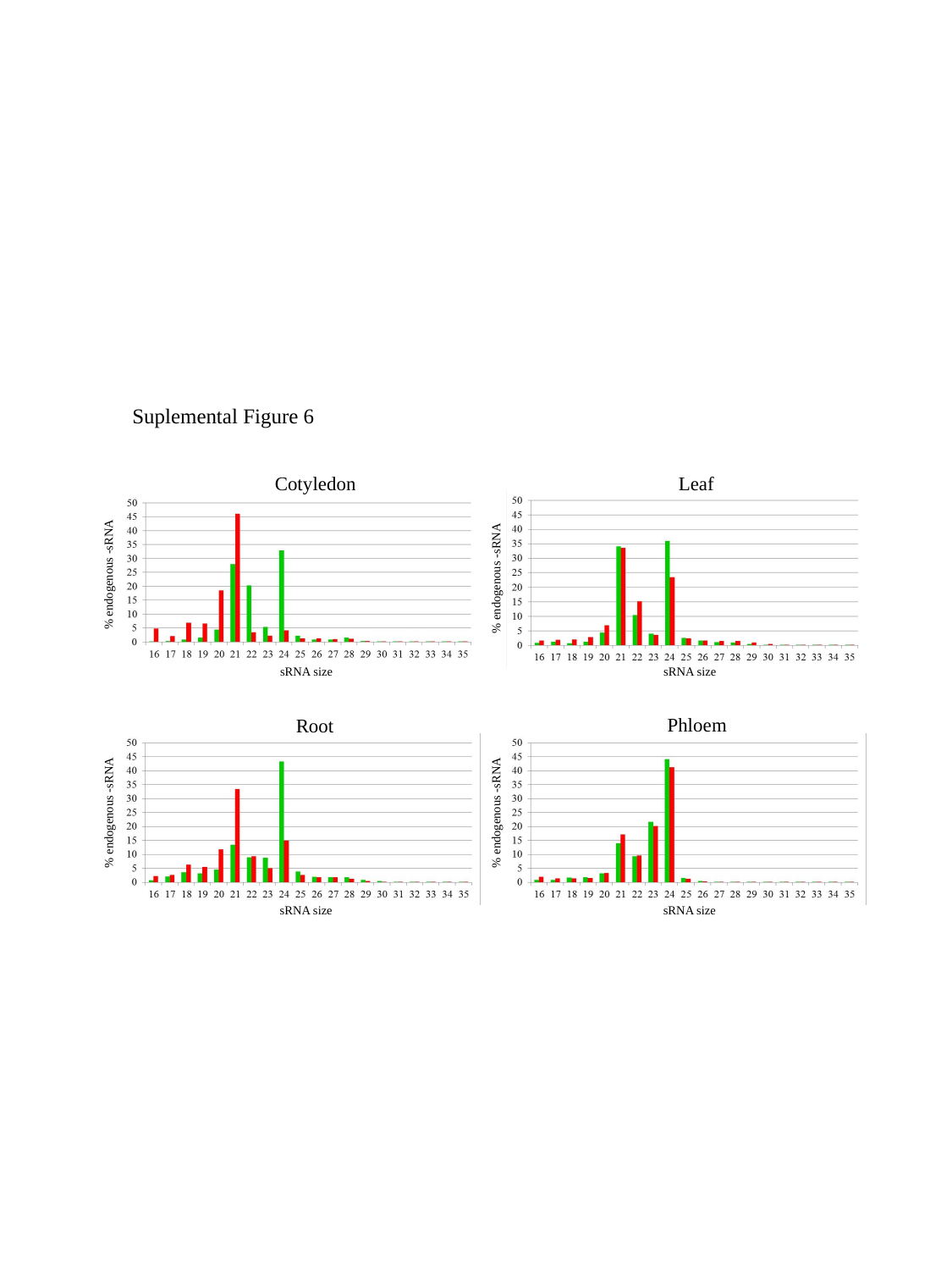

Suplemental Figure 6
Leaf
Cotyledon
% endogenous -sRNA
% endogenous -sRNA
sRNA size
sRNA size
Phloem
Root
% endogenous -sRNA
% endogenous -sRNA
sRNA size
sRNA size
